# Gestational Outcomes Related to the Occurrence of Gestational Diabetes Mellitus: A Cohort Study

**DOI:** 10.3390/healthcare12191905

**Published:** 2024-09-24

**Authors:** Samara Souza Stork, Claudia Meurer Souza, Josiane Somariva Prophiro, Elizabeth Ann Brownell, Betine Pinto Moehlecke Iser

**Affiliations:** 1 Postgraduate Program in Health Sciences, University of Southern Santa Catarina, Tubarão 88704-900, Brazil; samarastork@gmail.com (S.S.S.); claudia.meurer@hotmail.com (C.M.S.); josiane.prophiro@hotmail.com (J.S.P.); 2 School of Nursing, University of Texas Health, San Antonio, TX 78229, USA

**Keywords:** pregnancy, hyperglycemia, gestational diabetes mellitus, gestational complications

## Abstract

Background: Gestational diabetes mellitus (GDM) is the main cause of hyperglycemia in pregnancy and is related to complications throughout the gestational and post-partum period. Objectives: To analyze the pregnancy outcomes related to the occurrence of GDM in women and their offspring. Methods: Third-trimester pregnant women were interviewed and monitored until childbirth. The diagnosis of GDM, blood glucose ≥ 92 mg/dL, was defined by the criteria of the International Association of the Diabetes and Pregnancy Study Groups (IADPSG). Results: A total of 138 women participated, and there were 136 births (due to 2 fetal losses); 23 (16.7%) were diagnosed with GDM. The risk of complications during childbirth was higher among pregnant women with GDM (RR 3.40; 95%CI 1.65–7.00), as was the occurrence of cesarean birth (RR 1.9; 95%CI 1.46–2.59). The occurrence of preterm birth did not show a significant difference between GDM/non-GDM groups. There was a non-significant association in adjusted analyses of macrosomia (birth weight ≥ 4000 g) among newborns born to mothers with GDM (RR 1.27; 95%CI 0.67–2.38). For newborns born to pregnant women with GDM, there was a higher risk for the following outcomes: large for gestational age (LGA) (3.29 95%CI 1.62–6.64), low Apgar (4.98 95%CI 2.32–10.69), and birth asphyxia (9.51 95%CI 3.42–26.48). Conclusions: The findings reinforce that GDM is an important risk factor for adverse pregnancy outcomes for women and their offspring.

## 1. Introduction

Pregnancy is characterized as a physiological phenomenon that evolves uneventfully in most cases. However, a small proportion of women experience poor outcomes, diagnosed prenatally or perinatally, that contribute to increased maternal and fetal risk [1]. Further, the occurrence of maternal, fetal, and infant death is closely related to either or both of these pre-existing co-morbidities and/or those identified during pregnancy [1].

Among the occurrences, maternal hyperglycemia is associated with increased complications in pregnant women, such as systemic arterial hypertension, pre-eclampsia, cesarean birth, and neonatal complications [2]. This hyperglycemic condition can be detected in women with a previous diagnosis of diabetes mellitus (DM) or identified during pregnancy, defining the condition of gestational diabetes mellitus (GDM) [3].

GDM is the form of hyperglycemia with the highest prevalence during pregnancy, being defined by insulin resistance that begins during the gestational period in women without a previous diagnosis of DM and with blood glucose levels below diagnostic threshold parameters for DM [2,4,5]. No consensus exists regarding the screening and diagnostic methods for identifying GDM. However, the standard of care frequently applies the threshold proposed by the International Association of the Diabetes and Pregnancy Study Groups (IADPSG) (blood glucose ≥ 92 mg/dL) [6].

Various negative pregnancy outcomes associated with the occurrence of hyperglycemia and GDM include macrosomia and/or large for gestational age (LGA) babies [7], in addition to prematurity, hypoglycemia, intrauterine death, and respiratory dysfunction in the neonate [2]. Also, in the long term, women with GDM have an increased risk for the subsequent development of type 2 DM and cardiovascular diseases, and babies will have an increased risk of developing obesity and metabolic syndrome [3,8].

Accordingly, the increased risk of negative perinatal outcomes and the development of future co-morbidities resulting from GDM highlight the clinical and public health importance of the association between GDM and adverse gestational outcomes. These associations have been little explored in different regions of Brazil. Therefore, the present study aimed to analyze the gestational outcomes related to the occurrence of GDM in women and their descendants in a municipality in Southern Brazil.

## 2. Materials and Methods

### 2.1. Type of Study, Sample Characteristics, and Data Collection

The present research is characterized as an observational epidemiological prospective cohort study. The study population comprised pregnant women in the third trimester undergoing prenatal care by the Sistema Único de Saúde (SUS, Unified Health System) in a municipality in Southern Brazil, with a total population of 33,860 inhabitants and services distributed among 18 health units. The study population was recruited from December 2019 through March 2020, as described previously [9].

In the first phase of the study, pregnant women were invited to participate during their routine consultations at the Unidades Básicas de Saúde (UBS, Basic Health Units) of the municipality. After being informed about the study, those who agreed to participate signed the Free and Informed Consent Form (FICF), ensuring that participants are fully aware of and understand the nature, purpose, risks, and benefits of the study and confirming their voluntary agreement to participate. This process was essential for protecting participants’ rights and ensuring ethical standards in research. In this face-to-face contact with the pregnant woman, information was obtained about the socioeconomic level, assessed using the Brazilian Economic Classification Criterion from the Associação Brasileira de Empresas de Pesquisas (ABEP, Brazilian Association of Research Companies), and other personal characteristics (such as name, age, and preferred contact), in a questionnaire structured by the main author. Pregnant women who reported a previous diagnosis of DM; who withdrew from participating or from responding to the questionnaire during its first application; and who had psychiatric, cognitive, or other diseases that could adversely affect the understanding of the study objectives or the ability to provide accurate information were excluded. Furthermore, those who withdrew from participating in the research during pregnancy follow-up, did not provide the necessary information, or whose birth data were not found were not included in follow-up analyses. More details about the methods can be found in a previous publication [9].

GDM status was assessed based on the fasting blood glucose test, generally performed before the 20th week of pregnancy, or the Oral Glucose Tolerance Test (OGTT), measured three times, performed between the 20th and 28th weeks. This study included the number of women with blood glucose ≥ 92 mg/dL and ≤125 mg/dL for the analysis of the prevalence of GDM [2,10].

Data regarding pregnancy complications were abstracted from the electronic medical records of the pregnant women. To monitor the pregnancy and assess its outcomes, a protocol of weekly regular contact with the participants was followed through phone calls or messages via the WhatsApp^®^ application, in addition to consulting the electronic medical record via the system and contact with health teams to identify the period of birth of the babies. Pregnant women were monitored up to 10 days postpartum. Gestational outcomes and postpartum characteristics (fetal loss, macrosomia, gestational age at birth, newborn weight, neonatal hypoglycemia, and Apgar score) were evaluated in postpartum follow-up consultations, medical records, and/or obtained by telephone contact with the mothers.

### 2.2. Statistical Analysis

Data were entered in the Microsoft Excel 2011 program and exported for statistical analysis in SPSS software version 21.0 and Stata 16.0. Measures of the central tendency and dispersion were used to describe the quantitative variables. Qualitative variables were described in absolute (n) and relative (%) frequencies.

The variables were categorized as follows: for birth weight, low weight (<2500 g), adequate (≥2500 g and <3999 g), and macrosomia (≥4000 g) were considered. For gestational age: small for gestational age (SGA)—below the 10th percentile, appropriate for gestational age (AGA)—between the 10th and 90th percentiles, and LGA—above the 90th percentile [7] were considered. For Apgar classification: without asphyxia—Apgar 8 to 10, and with asphyxia, when Apgar ≤ 7, were considered. The dataset can be accessed in the Supplementary Material.

To verify the association between the variables of interest, Pearson’s chi-square test estimated differences in categorical variables, and Student’s *t* test evaluated differences in continuous variables; non-parametric correspondents were used as needed based on the identification of heteroscedasticity. The primary exposure was the presence of GDM as a risk factor for adverse gestational outcomes at birth and birth. The variables with *p* values < 0.20 in the binary analysis were submitted to multivariate models of adjustment of the estimates by robust Poisson regression model in Stata 16.0 software, and the results were expressed as a risk ratio (RR) with a 95% confidence interval (CI).

The present research followed Resolution 466/2012 of the National Health Council of Brazil and was approved by the Research Ethics Committee of the University under opinion number 3,395,300.

## 3. Results

First, 161 women in the third trimester of pregnancy were identified. Of these, 141 responded to the questionnaire. However, during the follow-up, there were 3 dropouts, totaling a sample of 138 pregnant women. The three participants withdrew their consent, and their data were excluded from the study analysis. Table 1 presents the main clinical characteristics of pregnant women and subsequent birth outcomes. There were no multiple births in the study population. The frequency of pregnant women who were diagnosed with GDM in the prenatal period, according to the cutoff point suggested by the IADPSG6, was 16.7% (n = 23). The comparison of the characteristics of women according to the diagnosis of GDM was presented previously [9].

The average age among the evaluated pregnant women was 27.21 years (SD [standard deviation] ± 6.14), ranging from 14 to 42 years old. Regarding birth outcomes, the most common complications were high blood pressure (5.1%), premature birth (3.7%), and hyperglycemia (2.9%). Five (3.7%) women had premature births between 34 and 36 weeks, and in one of the cases, the pregnant woman was diagnosed with GDM. The mean gestational age at birth was 38.97 weeks (SD ± 1.10).

Regarding neonatal characteristics, due to two fetal losses, one due to undiagnosed pre-eclampsia, and another due to cardiac malformation, 136 newborns were included in the analyses. The newborns had a mean birth weight of 3431 kg (SD ± 486.7) and a length of 48.5 cm (SD ± 2.48), with 16.9% presenting macrosomia and 19.1% being LGA. Newborn complications were observed in 7.4%: neonatal hypoglycemia (3.7%), followed by low oxygen (1.5%) and hydrocephalus in one case (0.7%). Considering those women already diagnosed with GDM in Basic Health Units, only seven (58.3%) had reached the standard of glucose levels, mainly by diet, as already described [9]. Among those considered uncontrolled, four had some intercurrence in newborns: four macrosomia and two hypoglycemia.

Regarding the monitoring of the NB and the presence of hyperglycemia during pregnancy, maternal GDM was associated with an increased risk of macrosomia, LGA NB, neonatal hypoglycemia, low 1st- and 5th-minute Apgar, and the occurrence of neonatal asphyxia (Table 2).

In an adjusted analysis, GDM, together with complications during childbirth, was a factor related to NB complications. For macrosomia, GDM was not an independently associated factor (RR 1.27; 95%CI 0.67–2.38). GDM was an independent risk factor for LGA (3.29 95%CI 1.62–6.64), for low 1st- and 5th-minute Apgar scores (4.98 95%CI 2.32–10.69; 17.73 95%CI 4.73–66.5, respectively) and the occurrence of neonatal asphyxia (9.51 95%CI 3.42–26.48) (Table 2).

## 4. Discussion

GDM was observed in 16.7% of the studied population of pregnant women in Southern Brazil. This finding falls within the Brazilian Society of Diabetes estimates that 3–25% of pregnancies are affected by this condition, even when accounting for variation among ethnic groups and different diagnostic criteria [2]. Martins et al. [11] reported similar data in a study conducted in Brazil on the prevalence of GDM, with 18.5% of the evaluated pregnant women presenting GDM according to the same diagnostic criteria of this study, according to the cutoff point suggested by the IADPSG.

As for gestational complications, GDM was positively associated with the occurrence of cesarean births and complications in the birth of pregnant women (high blood pressure, premature birth, and hyperglycemia), as well as complications in the NB, the main ones being neonatal hypoglycemia, LGA babies and low Apgar. Similar data, relating GDM with these same complications in childbirth and the NB, have been previously reported [12,13,14].

The risk of complications in childbirth was higher among pregnant women with GDM when compared to pregnant women without a diagnosis of the disease. When assessing the type of birth, it was observed that GDM reduced the frequency of vaginal birth by around 70%, showing a significantly higher prevalence of cesarean births among the group of women with GDM. These findings are similar to those reported by Yue et al. [15], who identified an increased risk for cesarean birth among women diagnosed with GDM. The current findings show that GDM often leads to cesarean sections due to complications like fetal macrosomia and maternal hypertension. However, maternal obesity and pre-existing health conditions may also influence these outcomes, suggesting that a broader approach is needed in GDM management [13,14]. The higher occurrence of cesarean births may be related to the risk of pregnancy itself, which may be a risk factor for cesarean birth [16]. Consistently, the study by Martins et al. [11] reported that women with GDM had a higher incidence of hypertensive disorders and preterm birth compared to those without GDM, with preterm births occurring in approximately 3.5% of cases. Additionally, hyperglycemia was noted as a significant risk factor for adverse birth outcomes. This comparison underscores the association between GDM and increased risk of complications such as preterm birth and high blood pressure.

As for gestational age at birth, when assessing the frequency of preterm births, there was no statistically significant difference between the group of pregnant women with and without GDM. These observed findings are inconsistent with those reported by Yue et al. [15], who reported an increased risk of preterm birth among pregnant women with GDM, which may be related to the greater number of the sample evaluated in the latter study.

As for NB complications, the current study also found an increased risk with the presence of GDM, which is in agreement with Fuka et al. [17] and Silva et al. [18]. The characteristics of LGA newborns and neonatal hypoglycemia, observed in this study with a significant risk among pregnant women with GDM, are consistent with data reported in the literature [7,19,20,21,22].

For Apgar and neonatal asphyxia, there are few studies evaluating the relationship with GDM. Hildén et al. [23] identified a slightly increased risk of low Apgar scores in descendants of women with GDM compared to women without the diagnosis of the disease. Yeaglo et al. [24] did not observe a significant association between low 1st- and 5th-minute Apgar scores and GDM. Fuka et al. [17] reported that low 5th-minute Apgar scores in newborns born to pregnant women with GDM were associated with gestational age at birth (<37 weeks) and maternal pre-eclampsia.

When evaluating the data for adequate birth weight, a higher prevalence of macrosomic newborns was identified among the group of pregnant women diagnosed with GDM (65.2%) when compared to newborns born to pregnant women without the disease (36.4%). Besides that, all five women with GDM whose glycemia was not controlled had macrosomic babies. However, this association was attenuated after adjusted analyses; the macrosomia in the NB was no longer statistically associated with maternal GDM status, consistent with previous research [15]. The data observed in this and other studies suggest that maternal weight has a greater influence on macrosomia of the NB, than GDM [7,25].

Adverse outcomes of GDM, such as increased cesarean deliveries and neonatal complications, are widely recognized and reflect findings in the literature. However, additional factors such as maternal obesity and pre-existing health conditions also play a significant role and may exacerbate these risks. This highlights the need to consider these additional variables when managing pregnancies complicated by the disease.

We must consider that the results of the diagnostic tests had already occurred before the beginning of the follow-up, and the monitoring was carried out from the third trimester until one month after the birth. Because of this, the analysis of conditions that had been already presented before the beginning of the study is limited. This study’s small sample size and the low prevalence of some conditions reduced the precision of the investigated exposure–outcome association. This limitation can be attributed to several factors: the relatively small population size of the study area, which constrained the number of available participants, and potential biases introduced by incomplete data on medical registries. We could not consider the type of therapy and glycemic control in the analysis of associations due to the small number of women diagnosed and, as such, eligible for treatment. Additionally, the narrow scope of the study may have affected the generalizability of the findings. Addressing these issues in future research will be crucial for enhancing the robustness and applicability of the results.

It is noteworthy that this study addresses the issue according to the reality of public health services in Brazil, as pregnant women were approached during routine prenatal consultations, therefore not including women who did not undergo consultations in the Primary Health Care system. It is also worth noting that this study focuses specifically on the southern region of Brazil, acknowledging the country’s vast territorial expanse and the significant cultural and socioeconomic differences across its regions. Given these disparities, we cannot guarantee the generalizability of our findings to different scenarios and populations.

## 5. Conclusions

In conclusion, the analysis of perinatal complications in women and newborns born in the group diagnosed with GDM demonstrated a significant risk between the adverse outcomes found in the study and the diagnostic status of these women during the gestational period. This study found that complications such as high blood pressure, premature birth, and hyperglycemia were notably prevalent among this group. These findings underscore the critical need for heightened awareness among women and healthcare providers regarding the serious implications of GDM. Increased education about the risks and management of GDM is essential to improve maternal and neonatal outcomes.

To address these challenges, there is a pressing need for standardized diagnostic approaches and more effective treatment strategies. Implementing consistent screening protocols and preventive measures, such as lifestyle modifications and regular monitoring, can significantly mitigate the risks associated with GDM. Future research should focus on optimizing diagnostic criteria, exploring novel treatment options, and assessing the long-term impact of preventive interventions. Collaborative efforts between clinicians and researchers are vital to advancing the understanding and management of GDM, ultimately improving health outcomes for mothers and their newborns.

## Figures and Tables

**Table 1:** Clinical characteristics of third-trimester pregnant women and evolution of pregnancy in a municipality in Southern Brazil, 2019. N = 138.

Characteristics	Total Sample n (%)
Pre-gestational BMI ^a^	
Low	7 (5.1)
Eutrophy	64 (46.4)
Overweight	67 (48.5)
DM family history	
Yes	75 (54.3)
No	63 (45.7)
Illness prior to pregnancy	
Yes	27 (19.6)
No	111 (80.4)
Weight gain during pregnancy *^b^	
Low	11 (8.1)
Adequate	69 (50.7)
Excessive	56 (41.2)
Intercurrence in pregnancy	
Yes	85 (61.6)
No	53 (38.4)
Type of birth *	
Cesarean	67 (49.3)
Vaginal	69 (50.7)
Intercurrence in childbirth *	
Yes	22 (16.2)
No	114 (83.8)

^a^ Pre-gestational BMI: low weight (<18.5 kg/m^2^), adequate weight (from 18.5 to 24.9 kg/m^2^), overweight (25 to 29.9 kg/m^2^), and obesity (>30 kg/m^2^). ^b^ Weight gain during pregnancy was evaluated according to pre-pregnancy BMI: low weight (gain up to 18 kg), adequate weight (gain between 11.5 and 16 kg), overweight (gain between 7 to 11.5 kg), and obesity (gain up to 7 kg). * Data were from 136 pregnant women due to 2 sample losses resulting from fetal deaths.

**Table 2:** Gestational diabetes mellitus and its relationship with childbirth outcomes and newborns in a municipality in Southern Brazil, 2019. N = 136.

Characteristics	GDM ^a^					
	Yes % (n = 23)	No % (n = 113)	RR (95%CI)	*p*-Value	aRR ^b^ (95%CI)	*p*-Value
Pregnant Woman’s Data						
Type of Birth						
Cesarean	82.6	42.5	1.95 (1.46–2.59)	<0.001	1.51 (1.11–2.06)	0.009
Vaginal	17.4	57.5	--			
Intercurrence in Childbirth *						
Yes	39.1	11.5	3.40 (1.65–7.00)	0.003	3.05 (1.39–6.68)	0.005
No	60.9	88.5	--			
Gestational Age at Birth						
Premature birth	4.3	0.9	4.91 (0.32–75.73)	0.311	1.18 (0.70–1.95)	0.531
Term birth	95.7	99.1	--			
Newborn’s Data *						
Adequacy at Birth Weight						
Macrosomia	65.2	36.3	1.80 (1.22–2.64)	0.010	1.27 (0.67–2.38)	0.464
Low weight and adequate weight	34.8	63.7	--			
Gestational Age Classification ^c^						
LGA	52.2	12.5	4.17 (2.23–7.81)	<0.001	3.29 (1.62–6.64)	0.001
SGA and AGA	47.8	87.5	--			
Intercurrence in Newborn						
Yes	34.8	1.8	19.65 (4.46–86.61)	<0.001 ^#^	135.82 (27.35–674.35)	<0.001
No	65.2	98.2	--			
NB Hypoglycemia						
Yes	17.4	0.9	19.65 (2.30–167.9)	0.003 ^#^	127.32 (3.44–4704.32)	0.008
No	82.6	99.1	--			
Apgar 1st min.						
Low	52.2	9.7	5.36 (2.70–10.63)	<0.001 ^#^	4.98 (2.32–10.69)	<0.001
Normal	47.8	90.3	--			
Apgar 5th min.						
Low	30.4	2.7	11.46 (3.20–41.07)	<0.001 ^#^	17.73 (4.73–66.51)	<0.001
Normal	69.4	97.3				
Apgar Rating						
With asphyxia	39.1	5.3	7.37 (2.91–18.69)	<0.001 ^#^	9.51 (3.42–26.48)	<0.001
Without asphyxia	60.9	94.7	--			

^#^ Fisher’s exact test. RR = Risk Ratio. ^a^ GDM, gestational diabetes mellitus. ^b^ data adjusted for maternal age, gestational age at birth, pre-gestational BMI, and weight gain during pregnancy by robust Poisson regression model. ^c^ LAG, large for gestational age; SGA, small for gestational age; AGA, appropriate for gestational age. Note: The analyzed outcomes are shown in the rows, and the predictor factor of interest to the study, the presence of GDM, is in the columns. * Data were from 136 pregnant women due to 2 sample losses resulting from fetal deaths.

## Data Availability

The dataset from the original study can be accessed at: https://drive.google.com/drive/folders/10ktremBOrld4y2iuCP2MljsOAem-D2Jx?usp=drive_link (accessed on 19 July 2024).

## Supplementary Materials

The following supporting information (dataset) can be downloaded at: https://drive.google.com/drive/folders/10ktremBOrld4y2iuCP2MljsOAem-D2Jx?usp=drive_link (accessed on 19 July 2024).
